# Day-Night Variations in the Concentration of Neurotransmitters in the Rat Lumbar Spinal Cord

**DOI:** 10.5334/jcr.215

**Published:** 2021-07-19

**Authors:** Beatriz Shantal Jiménez-Zárate, Celia Piña-Leyva, Marina Rodríguez-Sánchez, Benjamín Florán-Garduño, Luis Antonio Jiménez-Zamudio, Ismael Jiménez-Estrada

**Affiliations:** 1 Department of Physiology, Biophysics and Neurosciences, CINVESTAV, IPN, México; 2 Department of Physiology, ENCB, IPN, México City, México; 3 Department of Pharmacology, CINVESTAV, IPN, México; 4 Department of Immunology, ENCB, IPN, México City, México

**Keywords:** Neurotransmitter concentration, Spinal cord, Circadian rhythms, Lumbar

## Abstract

The purpose of this study was to analyze the light-dark variations in the concentrations of several neurotransmitters in the lumbar spinal cord of rats. Six groups of male Wistar rats were exposed to a 12 h light-12 h dark cycle for 70 days. At different time points of the experimental day (8, 12, 16, 20, 24 and 4 h), one of the groups of rats was randomly selected to be sacrificed, and the spinal cords were removed. The gamma-aminobutyric acid (GABA), glutamate (GLU), dopamine, serotonin, epinephrine (E), and norepinephrine (NE) levels in each extracted spinal cord were measured with high-pressure liquid chromatography (HPLC)-EQ and HPLC-fluorescence systems. Our results indicate that the spinal concentrations of GABA and GLU showed sinusoidal variation in a 24 h cycle, with the highest peak in the dark period (~20 h). Dopamine and serotonin also fluctuated in concentration but peaked in the light period (between 8 and 12 h), while E and NE concentrations showed no significant fluctuations. The possible relationship between neurotransmitter spinal concentration and sensitivity to pain and locomotor activity is discussed. It was concluded that most of the neurotransmitter levels in the lumbar spinal cord showed circadian fluctuations coupled to a light-dark cycle.

## 1. Introduction

Circadian rhythmicity is inherent to all organisms and is one of the main characteristics of biological systems. It is present in most biochemical and molecular processes, from the transcription of genes in cellular nuclei to the control of the cell cycle [1], and it participates considerably in physiological and behavioral mechanisms. In mammals, the suprachiasmatic nucleus (SCN) located in the anterior hypothalamus of the brain is considered the central circadian regulatory nucleus that responds to the environmental light-dark cycle [2]. The core mechanism of circadian rhythmicity in the SCN is a transcriptional-translational feedback loop of a set of clock genes [3].

Although the SCN is crucial for the generation of biological rhythms in mammals, the expression and regulation of clock genes are not unique to that nucleus. The rhythmic expression of the same clock genes that regulate the oscillator in the SCN is widely distributed among other areas of the brain and the spinal cord [4] and many peripheral cells and tissues, including the liver, intestine, heart, and retina [5,6].

It is well established that communication between neuronal circuits in the central nervous system is based on the interaction of diverse inhibitory, excitatory and modulatory synaptic influences; these influences are mediated by a series of neurotransmitters and neuromodulators. In many neuronal nuclei and centers in the brain, it has been demonstrated that several neurotransmitters show rhythmic variations in concentration [6], but there is practically no experimental evidence about possible day-night fluctuations in neurotransmitter levels in the spinal cord. Knowledge of such variations coupled to a light-dark cycle could be of utmost importance for understanding the expression of sensory, nociceptive, proprioceptive, and motor processes in the spinal cord. In this study, we analyzed circadian variations in the level of several neurotransmitters (glutamate, GLU [excitatory, 7]; gamma-aminobutyric acid, GABA [inhibitory, 7]; dopamine, DA [inhibitory transmitter at the spinal level, 8]); serotonin, SER [mostly inhibitory, 9]; epinephrine, E and norepinephrine, NE [both facilitate inhibitory and excitatory transmission in the spinal cord, 10,11]) during a 12 h dark-12 h light cycle in the lumbar spinal cord of rats.

## 2. Materials and methods

### 2.1 Animals and experimental design

Male Wistar rats, provided by our institutional animal house, were accommodated in individual acrylic cages with ad libitum access to food and water and were maintained under a 12:12 h light-dark cycle (lights on at 6:00 AM) and controlled temperature (22 ± 1°C). At the age of 70 days, rats were sorted into six groups (n = 10 animals per group) and each group was randomly selected for sacrifice by cervical dislocation at 08:00, 12:00, 16:00, 20:00, 24:00 and 04:00 hours, day time, and subsequently decapitated. Then, their lumbar spinal cord was extracted by negative pressure injection of saline applied with a 20 ml-syringe through the vertebral central canal at the spinal sacral level (S2-S3). Experiments were conducted following the guidelines of the Mexican Official Norm (NOM-062-ZOO-1999) and National Institutes of Health Guide NIH Publication No. 8023 (revised in 1996) for the Care and Use of Laboratory Animals and approved by the Institutional Bioethical Committee for Care and Handling of Laboratory Animals (UPEAL-Protocol 013-02, CINVESTAV).

### 2.2 Determination of endogenous amino acids (GLU and GABA) in the rat spinal cord

Samples of each spinal cord extract (10 mg) were subjected to sonication for 30 seconds, with an amplification of 30%, homogenized with 200 µL of 30% methanol and centrifuged at 3,500 rpm for 5 min. The pellets were washed and resuspended in 1 N NaOH, and the amount of total protein in each sample was determined by the Bradford method.

The supernatant was analyzed with a high-pressure liquid chromatography (HPLC) system with electrochemical detection (ECD, Intro Antec Leyden). The separation of GABA (inhibitory neurotransmitter) and GLU (excitatory neurotransmitter) was achieved with a 2.1 × 50 mm dC18 column (Atlantis, Waters; mobile phase: 100 mM disodium hydrogen phosphate, 20% methanol, 3.5% acetonitrile, pH 6.7, adjusted with phosphoric acid). Then, each neurotransmitter was measured by precolumn derivation and ECD. Briefly, a derivation was achieved by mixing 12 µL of working derivation reagent (6.75 mg OPA, 2.5% methanol, 1.25 µL 2-b-mercaptoethanol and 97.5% 0.1 M sodium tetraborate buffer) with 30 µL of filtered supernatant (nylon membrane/0.45 µm pore size). The derivation was detected with a glassy carbon electrode (VT-03 Antec Leyden) set at ± 550 mV.

### 2.3 Determination of endogenous catecholamines (DA, SER, E and NE) in the rat spinal cord

The spinal cord tissue was homogenized in 200 µL of perchloric acid per sample and centrifuged at 3,500 rpm for 5 min. The pellets were washed and resuspended in 1 N NaOH, and the amount of total protein in each sample was determined by the Bradford method. The catecholamine concentration in the sample supernatant was analyzed by HPLC and fluorescence detection (excitation/emission = 279/320 nm, Waters 2475 Multiλ Fluorescence Detector, Waters Corporation). The samples were passed through a C18 column (Supelco, Sigma Aldrich). The mobile phase was 9.48 mg/L monochloroacetic acid, 189 mg/L EDTA, 166 mg/L 1-octane sulfonic acid, and 4.5% acetonitrile, pH 3.2, adjusted with NaOH. The chromatograms were processed by using Empower Software, USA.

### 2.4 Data analysis

Data are expressed as the amount of neurotransmitter (ng) per milligram of protein present in tissue sample extracts. The concentration of each neurotransmitter per hour was averaged (±S.E.M., n = 10). Rhythmic oscillation parameters (acrophase, mesor, zero-amplitude and percentage of rhythmicity) were determined with the cosinor test by using R software, v.3.6, which established the best adjustment of data to a sinusoidal waveform. Because the cosinor test adjusted the experimental data to a sinusoidal waveform, we decided to use the F test and Pearson’s correlation coefficient (r) to assess the fitness of the sinusoidal curve with the experimentally obtained concentration values of each neurotransmitter. In addition, to establish a possible synchronization between the day-night variations in the concentration of the different neurotransmitters analyzed, Pearson’s correlation coefficient was calculated between pairs of neurotransmitters. For graphic construction, we used the data processed with Chronos Fit software [12]. Differences between time points were analyzed by one-way ANOVA, followed by the Student-Newman-Keuls post hoc multiple comparison analysis method (Sygma plot v12.0, Systat Software Inc.). In all cases, statistical significance was set at p < 0.05.

## 3. Results

### 3.1 Variations in the concentration of spinal GABA and glutamate during the day-night cycle

**Figure 1:**
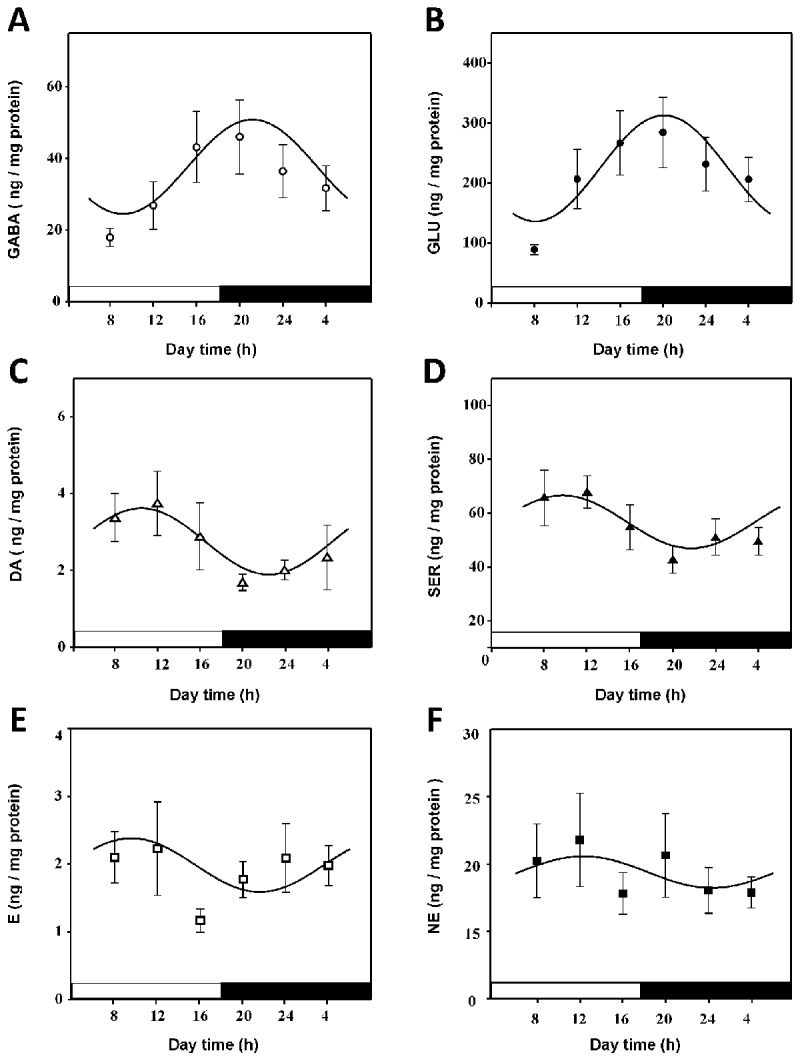
Changes in neurotransmitter levels in the lumbar spinal cord of rats during a light-dark cycle. **A**) Gamma-aminobutyric acid (GABA; open circles), **B**) glutamate (GLU; closed circles), **C**) dopamine (DA; open triangles), **D**) serotonin (SER; closed triangles), **E**) epinephrine (E; open squares) and **F**) norepinephrine (NE; closed squares). Values are the mean ± S.E.M. for 8–10 rats. Cosinor fitting was signiﬁcant in the GABA, GLU, DA, and SER concentration curves and experimental data (F test, p < 0.001) but not for the E and NE curves. The bar on the X axis indicates the light and dark periods.

GABA and glutamate in the lumbar spinal cord showed fluctuations in their levels that followed a sinusoidal curve during a light-dark cycle (F test, p < 0.02; r_(GABA)_ = 0.87 and r_(GLU)_ = 0.88, respectively; ***Figure 1A***). The level of GABA began to increase at the middle part of the light phase and reached its maximal value in the dark phase at ~20:00 h, almost one hour after the lights went off, while the lowest level of GABA was determined at the 08:00 h time point (***Figure 1A***). The mesor and zero-amplitude values were 33.68 and 26.02, respectively.

When comparing the GABA levels between time points, a significant difference between the values obtained at 08:00 and 20:00 h was found (one-way ANOVA, p < 0.05, Student-Newman-Keuls post hoc test). In the case of GLU (***Figure 1B***), variations in the detected level adjusted well to a 24 h sinusoidal curve (F test, p < 0.01; r_GLU_ = 0.81), which was similar to that of GABA (***Figure 1A***). The highest value was obtained at 20:00 h, and the lowest level was obtained at 08:00 h. GLU had an acrophase value of 19:31 h and mesor and zero-amplitude values of 214.4 and 160.6, respectively. Significant differences in GLU levels were determined between 08:00 h and 16:00 h and between 08:00 h and 20:00 h (one-way ANOVA, p < 0.05, Student-Newman-Keuls post hoc test). These results indicate that the concentration of GABA and GLU in the spinal cord varied during the light-dark cycle.

### 3.2 Fluctuation of the spinal concentration of catecholamines during the light-dark cycle

DA and SER followed a similar sinusoidal function (r_DA_ = 0.96 and r_SER_ = 0.91; P = 0.037, cosinor function; ***Figure 1C*** and ***1D***). The DA concentration reached its highest level during the light phase at 12:00 h, while the lowest level was observed at 20:00 h (***Figure 1C***). The rhythmic parameter acrophase reached a value of 2:44, and a mesor value of 2.67 and zero-amplitude of 2.04 was determined. Significant differences in DA levels were found between time points 12:00 h and 20:00 h (one-way ANOVA, p < 0.05, Student-Newman-Keuls post hoc test).

In the case of SER, the variation in its level in the spinal cord during a 12:12 light-dark cycle also followed a sinusoidal function (F, p < 0.02, r_(SER)_ = 0.91; ***Figure 1D***). With the acrophase at 10:18 h, almost four hours before the lights turned on, a mesor value of 55.06 and zero-amplitude of 23.72 were observed. These data indicated that the levels of DA and SER in the spine also showed a circadian variation of almost 24 h, but they appear to differ with respect to GABA and GLU sinusoidal curves by approximately 12 h (***Figure 1A*** and ***1B***). In contrast, the levels of E and NE in the spine showed no significant variations during the light-dark cycle (r_E_ = 0.35 and r_NE_ = 0.53; F test, p = 0.60 and p = 0.70, respectively), and as a consequence, they did not follow any sinusoidal variation.

### 3.3 Synchronization of fluctuations in neurotransmitter levels in the spine during the day-night cycle

Due to the time course similarities shown by the changes in the levels of GABA and GLU, DA and SER, but not E and NE, it is assumed that these neurotransmitters are correlated and highly synchronized. To explore the latter concept in this study, we determined the Pearson’s coefficient of correlation (r) of the averaged light-night variations in the levels of the aforementioned pairs of neurotransmitters in the spine in this study. ***Table 1*** shows the r values calculated for all pairs of neurotransmitters analyzed. Only GABA-GLU and DA-SER pairs showed the largest r values (r > 0.9), while the rest of the neurotransmitter pairs had relatively smaller r values (r < 0.6). These results could suggest that the levels of GABA-GLU and DA-SER neurotransmitter pairs fluctuate in a synchronized manner in the spine during a day-night period.

**Table 1:** Pearson’s coefficient of correlation values (r) for the concentration of pairs of neurotransmitters in the lumbar spinal cord of rats.


	GABA	GLU	DA	SER	E	NE

**GABA**		0.95	0.56	0.55	0.6	0.33

**GLU**	0.95		0.47	0.68	0.51	0.2

**DA**	0.56	0.47		0.93	0.3	0.52

**SER**	0.55	0.68	0.93		0.44	0.45

**E**	0.6	0.51	0.3	0.44		0.6

**NE**	0.33	0.2	0.52	0.45	0.6	


## 4. Discussion

In this study, we found that the level of GABA, GLU, DA, and SER in the lumbar spinal cord appears to fluctuate in a sinusoidal oscillation during a 12:12 light-dark cycle, while the levels of E and NE do not seem to fluctuate in a circadian day-night cycle. GLU and GABA are two of the most abundant neurotransmitters in the central nervous system [7]. Several studies have reported the presence of circadian rhythms in the GLU system in the CNS, with a maximal nocturnal increase [13]. In fact, several studies have demonstrated circadian rhythmicity in the expression of proteins related to glutamatergic neurotransmission, including NMDA receptor subunits and glutamate transporters [14]. Such circadian rhythm changes could influence the GLU-glutamine metabolic cycle and the number of neurotransmitters released from glutamatergic synapses. Such influences could especially affect the primary afferent terminals ending in the spinal dorsal horn, and it could induce the variation in GLU concentration during a light-dark cycle (12:12), as evidenced in this study.

In the case of GABA, it has been reported that the number of GABA-A receptors in the cerebral cortex fluctuates rhythmically, with their maximum value occurring at night [15]. Most importantly, it has been demonstrated that GABA in the SCN has an essential role in the regulation of circadian rhythmicity and that the GABAergic system within the SCN region also exhibits circadian rhythmicity in rats [16,17].

Brain monoamines, such as DA and SER, are involved in the regulation of several essential physiological functions, including the circadian rhythm. It has been shown that DA receptors exert direct or indirect influences on circadian clock genes and proteins in several brain areas [18]. Schade and collaborators [19] reported that in the nuclei accumbens and striatum, DA levels present a circadian rhythm with a maximal diurnal increase. Since most dopaminergic projections to the spinal cord originate from the hypothalamic A11 region [20], a probable synchronization between DA synthesis in the A11 region and between DA synthesis and the accumulation of DA in the lumbar spinal cord could be expected. Such a possibility could occur because our results indicate that DA levels in the lumbar spinal cord fluctuate rhythmically during a light-dark cycle, with a maximal peak occurring during the light phase. In addition, and in concordance with our results, Clemens and collaborators [21] showed that tyrosine hydroxylase, a rate-limiting enzyme for the production of DA, presents higher levels during the day in comparison to levels present at night in the spinal cord. Similar to DA, SER levels vary rhythmically in the lumbar spinal cord across the light-dark cycle, demonstrating higher concentration values in the light period.

Serotonergic descending axons originate from several nuclei in the brainstem, particularly from the ventrolateral periaqueductal gray nucleus and the nucleus raphe magnus, whose activation induces an antinociceptive effect in the spinal cord [22].

It is well known that neuronal ensembles in the lumbar spinal cord participate in an ample repertoire of sensory and motor processes, such as sensorial integration, pain, voluntary and nonvoluntary movements, posture and control of locomotion [23]; in the sensory and motor processes where these neuronal ensembles are involved, the participation of neurotransmitter systems has particular relevance. In a previous study, it was determined that changes in forebrain SER levels during the light-dark transition covaried significantly with changes in the levels of alert waking, a behavioral measure of the time spent in active walking [24]. According to the latter, it is tempting to propose a possible relationship between the light-dark variability in the level of neurotransmitters in the spine and the circadian variations in pain sensitivity and locomotor activity.

In rats, sensitivity to pain is modulated by neuronal and neurotransmitter ensembles in the spinal dorsal horn [25]. The expression of these ensembles varies in a light-dark pattern, with a minimal sensitivity to pain at the transition from light to darkness and maximal sensitivity a few hours before the transition from darkness to light [26]. Meanwhile, locomotor activity is controlled by neural circuits (central pattern generators) located in intermediate and ventral regions of the spinal gray matter [27]. This activity increases during the dark phase and decreases in the light phase [28]. According to the latter, it could be assumed that both physiological processes, pain sensitivity and locomotor activity, are modulated by independent sets of neurons and neurotransmitters located in the dorsal and ventral spinal regions; these independent sets of neurons and transmitters seem to vary, but not in phase with the day-night cycle.

Since our results were obtained by using the entire lumbar spinal cord, which prevented us from discerning the occurrence of regional variations in the levels of neurotransmitters in the spine, we assume that the levels of neurotransmitters in the spine determined at each time point along a light-dark period was due to the sum of several sensory and motor neuronal processes that occur in the spinal cord at each time point. Therefore, we consider that it is not feasible to establish a close relationship between light-dark variations in the level of neurotransmitters of the entire spinal cord and variations in individual physiological processes or behaviors. To establish this relationship, it would be necessary to use an experimental paradigm different from the one used in the present study.

Regarding E and NE, to the best of our knowledge, there are practically no experimental reports in the literature about the circadian rhythmicity of E and NE in the spinal cord of mammals. Our results reveal that the spinal levels of these monoamine neurotransmitters do not have significant differences between time points during a light-dark cycle, which is indicative of the possible absence of circadian rhythmicity in the level of E and NE in the spine. However, as mentioned above, our results do not allow us to exclude the possibility of the activation of two different sensory and/or motor processes that use NE and E as neurotransmitters in such way that their algebraic sum may alter or cancel the light-dark variation in NE and E levels, as observed in this study. These processes would be located in the dorsal and ventral regions of the spinal cord, and occur in parallel but would be completely desynchronized (i.e., one increases the level of NE and E in the spine during the light phase and the other increases the level of NE and E during the dark phase). This possibility needs to be evaluated with experimental approaches other than those used in this study.

As pointed out in the Results section, the time course similarities and high correlation values for the changes in spinal concentrations of GABA and GLU and of DA and SER are notable. Due to the latter, temporal synchronization between the circadian clocks of both pairs of neurotransmitters could be proposed. It is possible that such temporal synchronization of neurotransmitter pairs could be related to the dynamic balance of excitation and inhibition that occurs in neuronal circuits, which has been considered essential for the proper functioning of the brain and spinal cord [29,30]. However, this proposal needs to be analyzed with other experimental approaches to disclose the relative importance of the variations in the neurotransmitter concentration for neuronal communication in the spinal cord.

Finally, it is important to highlight the need to evaluate the day-night variation in the expression of clock genes, neurotransmitter receptors, and transporters at the lumbar spinal cord level to characterize the possible role of genetic and synaptic mechanisms in the physiological function of each of the neurotransmitters analyzed; moreover, such an evaluation could also reveal the possible routes of interaction between different circadian rhythms, such as those between the nucleus accumbens and the spinal cord.

## 5. Conclusions

The results obtained in this study demonstrate that the spinal concentration of GLU, GABA, DA, and SER vary during the 24 h day-night period, indicating that they are subjected to synchronized circadian rhythms coupled to a light-dark cycle. In contrast, E and NE levels do not seem to follow a circadian oscillation. Further studies are needed to evaluate the functional role of these light-dark fluctuations in the level of neurotransmitters in the spine that may influence the essential activity of spinal neuronal ensembles and the expression of sensory and motor-related behaviors during a light-dark cycle.

## Additional File

The additional file for this article can be found as follows:

10.5334/jcr.215.s1 Supplemental Data. Data corresponding to the article: Day-night variations of neurotransmitters content in the rat lumbar spinal cord.

## References

[1] Borgs L, Beukelaers P, Vandenbosch R, Belachew S, Nguyen L, Malgrange B. Cell “circadian” cycle: new role for mammalian core clock genes. Cell Cycle. 2009; 8: 832–837. DOI: 10.4161/cc.8.6.7869 19221497

[2] Rozenblit-Susan, S, Chapnik N, Genzer Y, Froy O. Serotonin suppresses food anticipatory activity and synchronizes the food-entrainable oscillator during time-restricted feeding. Behav Brain Res SreeTest Content. 2016; 297: 150–154. DOI: 10.1016/j.bbr.2015.10.019 26467604

[3] Okamura H, Yamaguchi S, Yagita K. Molecular machinery of the circadian clock in mammals. Cell Tissue Res. 2002; 309: 47–56. DOI: 10.1007/s00441-002-0572-5 12111536

[4] Morioka N, Sugimoto T, Tokuhara M, Nakamura Y, Abe H, Hisaoka K, Nakata Y. Spinal astrocytes contribute to the circadian oscillation of glutamine synthase, cyclooxygenase-1 and clock genes in the lumbar spinal cord of mice. Neurochemistry International. 2012; 60: 817–826. DOI: 10.1016/j.neuint.2012.03.005 22446583

[5] Yamazaki S, Numano R, Abe M, Hida A, Takahashi R, Ueda M, Block GD, Sakaki Y, Menaker M, Tei H. Resetting central and peripheral circadian oscillators in transgenic rats. Science. 2000; 288: 682–685. DOI: 10.1126/science.288.5466.682 10784453

[6] Zylka MJ, Shearman LP, Weaver DR, Reppert SM. Three period homologs in mammals: differential light responses in the suprachiasmatic circadian clock and oscillating transcripts outside of brain. Neuron. 1998; 20: 1103–10. DOI: 10.1016/S0896-6273(00)80492-4 9655499

[7] Honma S, Katsuno Y, Shinohara K, Abe H, Honma K. Circadian rhythm and response to light of extracellular glutamate and aspartate in rat suprachiasmatic nucleus. Am J Physiol. 1996; 271: 579–85. DOI: 10.1016/j.neuint.2012.04.016 8853378

[8] Smith AD, Olson RJ, Justice JB, Jr. Quantitative microdialysis of dopamine in the striatum: effect of circadian variation. J Neurosci Methods. 1992; 44: 33–41. DOI: 10.1016/0165-0270(92)90111-P 1279321

[9] Rueter LE, Jacobs BL. Changes in forebrain serotonin at the light-dark transition: correlation with behaviour. Neuroreport. 1996; 7: 1107–11. DOI: 10.1097/00001756-199604100-00031 8804061

[10] Zuther S, Gorbey, Lemmer B. Chronos-Fit 1.06. 2009. http://www.ma.uni-heidelberg.de/inst/phar/lehre/chrono.html.

[11] Carlsson A, Svennerholm L, Winblad B. Seasonal and circadian monoamine variations in human brains examined post mortem. Acta Psych Scand. 1980; 280: 75–85. DOI: 10.1111/acps.1980.61.s280.75 6157305

[12] Wagner S, Castel M, Gainer H, Yarom Y. GABA in the mammalian suprachiasmatic nucleus and its role in diurnal rhythmicity. Nature. 1997; 387: 598–603. DOI: 10.1038/42468 9177347

[13] Castañeda TR, de Prado BM, Prieto D, Mora F. Circadian rhythms of dopamine, glutamate and GABA in the striatum and nucleus accumbens of the awake rat: modulation by light. J Pineal Res. 2004; 36: 177–185. DOI: 10.1046/j.1600-079X.2003.00114.x 15009508

[14] Marquez de Prado B, Castañeda TR, Galindo A, del Arco A, Segovia G, Reiter RJ, Mora F. Melatonin disrupts circadian rhythms of glutamate and GABA in the neostriatum of the awake rat: a microdialysis study. J Pineal Res. 2000; 29: 209–216. DOI: 10.1034/j.1600-0633.2002.290403.x 11068943

[15] Chi-Castañeda D, Ortega A. Circadian Regulation of Glutamate Transporters. Front. Endocrinology. 2018; 9: 340. DOI: 10.3389/fendo.2018.00340 PMC6021491 29977228

[16] Wang LM, Schroeder A, Loh D, Smith D, Lin K, Han JH, Michel S, Hummer DL, Ehlen JC, Albers HE, Colwell CS. Role for the NR2B subunit of the N-methyl-D-aspartate receptor in mediating light input to the circadian system. Eur J Neurosc. 2008; 27: 1771–1779. DOI: 10.1111/j.1460-9568.2008.06144.x PMC2586987 18380671

[17] Yao Z, DuBois DC, Almon RR, Jusko WJ. Modeling circadian rhythms of glucocorticoid receptor and glutamine synthetase expression in rat skeletal muscle. Pharm. Res. 2006; 23: 670–679. DOI: 10.1007/s11095-005-9608-3 16673181 PMC4178542

[18] Kanterewicz BI, Rosenstein RE, Golombek DA, Yannielli PC, Cardinali DP. Daily variations in GABA receptor function in Syrian hamster cerebral cortex. Neurosc lett. 1995; 200: 211–213. DOI: 10.1016/0304-3940(95)12112-H 9064614

[19] Aguilar-Roblero R, Verduzco-Carbajal L, Rodríguez C, Mendez-Franco J, Morán J, de la Mora MP. Circadian rhythmicity in the GABAergic system in the suprachiasmatic nuclei of the rat. Neurosc lett. 1993; 157: 199–202. DOI: 10.1016/0304-3940(93)90736-5 8233053

[20] Korshunov KS, Blakemore LJ, Trombley PQ. 2017. Dopamine: A Modulator of Circadian Rhythms in the Central Nervous System. Front Cellular Neurosc. 2017; 11: 91. DOI: 10.3389/fncel.2017.00091 PMC5376559 28420965

[21] Daut RA, Fonken LK. 2019. Circadian regulation of depression: A role for serotonin. Front Neuroendocrinology. 2019; 54: 100746. DOI: 10.1016/j.yfrne.2019.04.003 PMC9826732 31002895

[22] Schade R, Vick K, Ott T, Sohr R, Pfister C, Bellach J, Golor G, Lemmer B. Circadian rhythms of dopamine and cholecystokinin in nucleus accumbens and striatum of rats-influence on dopaminergic stimulation. Chronobiology Int. 1995; 12: 87–99. DOI: 10.3109/07420529509064504 8653803

[23] Puopolo M. The hypothalamic-spinal dopaminergic system: a target for pain modulation. Neural Regeneration Res. 2019; 14: 925–930. DOI: 10.4103/1673-5374.250567 PMC6404492 30761995

[24] Clemens S, Sawchuk MA, Hochman S. Reversal of the circadian expression of tyrosine-hydroxylase but not nitric oxide synthase levels in the spinal cord of dopamine D3 receptor knockout mice. Neuroscience. 2005; 133: 353–357. DOI: 10.1016/j.neuroscience.2005.03.002 15878801 PMC2705059

[25] Kwiat GC, Basbaum AI. The origin of brainstem noradrenergic and serotonergic projections to the spinal cord dorsal horn in the rat. Somatosensory Mot Res. 1992; 9: 157–173. DOI: 10.3109/08990229209144768 1354402

[26] Bican O, Minagar A, Pruitt AA. The spinal cord: a review of functional neuroanatomy. Neurologic Clinics. 2013; 31: 1–18. DOI: 10.1016/j.ncl.2012.09.009 23186894

[27] Todd AJ. Neuronal circuitry for pain processing in the dorsal horn. Nat Rev Neurosci. 2010; 11(12): 823–836. DOI: 10.1038/nrn2947 21068766 PMC3277941

[28] Christina A, Merlin N, Vijaya C, Jayaprakash S, Murugesh N. Daily rhythm of nociception in rats. J Circadian Rhythms. 2004; 2: 2. DOI: 10.1186/1740-3391-2-2 15043763 PMC395843

[29] Guertin P. Central pattern generator for locomotion: Anatomical, Physiological, and pathophysiological consideration. Front Neurol. 2012; 3: 183. DOI: 10.3389/fneur.2012.00183 23403923 PMC3567435

[30] Stephan FK, Zucker I. Circadian rhythms in drinking behavior and locomotor activity of rats are eliminated by hypothalamic lesions. Proc Nat Acad Sci. 1972; 69(6): 1583–1586. DOI: 10.1073/pnas.69.6.1583 4556464 PMC426753

